# MicroRNA-21 in Pancreatic Ductal Adenocarcinoma Tumor-Associated Fibroblasts Promotes Metastasis

**DOI:** 10.1371/journal.pone.0071978

**Published:** 2013-08-22

**Authors:** Brian E. Kadera, Luyi Li, Paul A. Toste, Nanping Wu, Curtis Adams, David W. Dawson, Timothy R. Donahue

**Affiliations:** 1 Division of General Surgery, Department of Surgery, David Geffen School of Medicine at University of California Los Angeles, Los Angeles, California, United States of America; 2 Department of Pathology and Laboratory Medicine, David Geffen School of Medicine at University of California Los Angeles, Los Angeles, California, United States of America; 3 Department of Molecular and Medical Pharmacology, David Geffen School of Medicine at University of California Los Angeles, Los Angeles, California, United States of America; 4 Jonsson Comprehensive Cancer Center, David Geffen School of Medicine at University of California Los Angeles, Los Angeles, California, United States of America; 5 Institute for Molecular Medicine, David Geffen School of Medicine at University of California Los Angeles, Los Angeles, California, United States of America; Sapporo Medical University, Japan

## Abstract

**Introduction:**

Pancreatic ductal adenocarcinoma (PDAC) is projected to rise to the second leading cause of U.S. cancer-related deaths by 2020. Novel therapeutic targets are desperately needed. MicroRNAs (miRs) are small noncoding RNAs that function by suppressing gene expression and are dysregulated in cancer. miR-21 is overexpressed in PDAC tumor cells (TC) and is associated with decreased survival, chemoresistance and invasion. Dysregulation of miR regulatory networks in PDAC tumor-associated fibroblasts (TAFs) have not been previously described. In this study, we show that miR-21 expression in TAFs promotes TC invasion.

**Methods:**

In-situ hybridization for miR-21 was performed on the 153 PDAC patient UCLA tissue microarray and 23 patient-matched lymph node metastases. Stromal and TC histoscores were correlated with clinicopathologic parameters by univariate and multivariate Cox regression. miR-21 positive cells were further characterized by immunofluorescence for mesenchymal/epithelial markers. For *in vitro* studies, TAFs were isolated from freshly resected human PDAC tumors by the outgrowth method. miR-21 was overexpressed/inhibited in fibroblasts and then co-cultured with GFP-MiaPaCa TCs to assess TC invasion in modified Boyden chambers.

**Results:**

miR-21 was upregulated in TAFs of 78% of tumors, and high miR-21 significantly correlated with decreased overall survival (P = 0.04). Stromal miR-21 expression was also significantly associated with lymph node invasion (P = 0.004), suggesting that it is driving TC spread. Co-immunofluorescence revealed that miR-21 colocalized with peritumoral fibroblasts expressing α-smooth muscle actin. Moreover, expression of miR-21 in primary TAFs correlated with miR-21 in TAFs from patient-matched LN metastases; evidence that PDAC tumor cells induce TAFs to express miR-21. miR-21 expression in TAFs and TCs promotes invasion of TCs and is inhibited with anti-miR-21.

**Conclusions:**

miR-21 expression in PDAC TAFs is associated with decreased overall survival and promotes TC invasion. Anti-miR-21 may represent a novel therapeutic strategy for dual targeting of both tumor and stroma in PDAC.

## Introduction

Pancreatic ductal adenocarcinoma (PDAC) is currently the fourth leading cause of cancer-related deaths in the United States [1]. If the current trends continue, it is predicted to rise to second behind lung cancer by 2020 [2]. This rising mortality can be prevented with earlier diagnosis or improved treatment strategies. Rapid autopsy evaluation of patients who died of PDAC revealed that over 70% had macrometastases, most commonly to the liver and then lung [3]. The large tumor-associated stromal volume and its components in PDAC are thought to be a major contributor of the propensity of this tumor to spread to distant organs [4]. A better understanding of how the stroma contributes to metastasis development in PDAC may lead to new treatment strategies that improve the prognosis of this fatal disease.

MicroRNAs (miRs) are small noncoding RNAs that are approximately 20 nucleotides in length [5]. Through complementary base-pairing, they bind the 3′UTR of their target mRNAs and silence their translation via the RISC complex. miRs can regulate the expression of many target genes and are associated with developmental processes and cancer [5]. Profiling of poorly differentiated solid tumors from multiple organs revealed that miRs are more cancer-specific than mRNAs [6]. Our previous study revealed that miRs in PDAC tumor cells (TC) are extensively involved in regulating expression of genes associated with survival [7].

miR-21 is expressed in many solid tumors including hepatocellular carcinoma [8], colon [9] and pancreatic cancers[10]–[14]. In PDAC TCs miR-21 expression (i) increases early during tumorigenesis in low grade premalignant pancreatic intraepithelial neoplasias (PanIN) [15]; (ii) mediates TC invasion, proliferation, and chemoresistance in cell culture [12]; and (iii) is associated with shorter overall survival in patients [11], [13]. However, miR-21′s pro-tumorigenic impact is not limited to the TC compartment, as expression of miR-21 in the stroma of colorectal cancers predicts shorter disease-free survival [16], [17].

Therefore, based on (i) the association of decreased survival and miR-21 expression in PDAC TCs and (ii) colon cancer stroma, and (iii) the pro-tumorigenic function of miR-21 in PDAC TCs, we hypothesized that miR-21 expression in the PDAC stroma enhances TC invasion and metastasis. Using human tumor samples and primary cell cultures, we find that PDAC TCs induce peritumoral fibroblasts to express miR-21, which promotes TC invasion. Inhibition of miR-21 in PDAC TCs and tumor-associated fibroblasts (TAFs) is additive in reducing TC invasion. These findings provide evidence that miR-21 may be a good dual TC and stromal cell anti-metastatic target for therapy and a novel strategy to improve the prognosis of this fatal disease.

## Methods

### Ethics Statement

This study was approved by the UCLA Institutional Review Board and the UCLA Office of Animal Research Oversight. Written consent was obtained from all patients.

### In Situ Hybridization for microRNA-21 and TMA Scoring

The UCLA tissue microarray (TMA) includes tumor cores for 153-patients (Table S1), all with well-annotated clinical histories, and has been previously described [18]. TMA slides or FFPE samples of primary PDACs were incubated at 60°C for 1 hour, deparaffinized in xylene, and rehydrated with graded alcohol washes. Slides were then washed 3 times with diethyl pyrocarbonate-treated PBS, digested with 5 µg/mL proteinase K at 37°C for 30 minutes, washed then dehydrated in graded alcohol. Slides were hybridized at 55°C for 2 hours with 50 nmol/L locked-nucleic acid (LNA)-modified DIG-labeled probes for miR-21 (Exiqon, Vedbæk, Denmark). After stringency washes (5×, 1×, 0.2×SSC), slides were placed in blocking solution for 1 hour at RT followed by overnight incubation at 4°C in alkaline phosphatase conjugated anti-DIG Fab fragment solution. Antibody signal was amplified with NBT and BCIP substrate (Roche, Mannheim, Germany) and then tissue was counterstained with Nuclear Fast Red (Vector Laboratories, Burlingame, CA). Each TMA core was scored by two independent M.D. observers for intensity of staining in TCs and stromal spindle shaped cells (likely fibroblasts) using the scale: 0 negative, 1 weakly positive, 2 moderately positive, and 3 strongly positive. When there was a discrepancy, a consensus score was determined by the 2 observers. The median score from the 3 separate cores for each tumor was used for categorization of high versus low miR-21 expression. Patients with greater than the median value (n = 73 of 145) were categorized as high miR-21. Eight cores were omitted due to poor tissue preservation.

### Immunofluorescence Staining

Upon rehydration as above, FFPE tumor samples were boiled in 0.01 M sodium citrate buffer for 15 minutes. After blocking for 1 hour with 5% donkey serum in PBS at RT, primary antibody was added to serial sections, α-SMA 1∶2500 (Sigma-Aldrich, St. Louis, MO), nestin 1∶100 (Abcam, Cambridge, UK) or vimentin 1∶100 (Cell Signaling Technology, Inc., Danvers, MA) and incubated at 4°C overnight. After washing, secondary antibody Alexa Fluor® 594 anti-mouse or Alexa Fluor® 488 anti-rabbit (Molecular Probes, Inc., Life Technologies Corp., Carlsbad, CA) 1∶1000 was incubated for 1 hour at RT. The slides were then mounted, counterstained with DAPI and visualized. For *in situ* immunofluorescence staining, cells were first grown to 80% confluency on coverslips, fixed and permeabilized with 4% paraformaldehyde and 0.5% Triton X-100 in PBS. Blocking and antibody incubation were then carried out as above with the inclusion of additional primary antibodies, GFAP 1∶1000 (DAKO, Glostrup, Denmark) and PanCK 1∶250 (Sigma). To create an overlay of immunofluorescence staining with miR-21 *in situ* hybridization (ISH), the ISH image was converted to a digital negative and then serial sections were combined using Photoshop® CS6 (Adobe Systems Inc., San Jose, CA).

### Cell Culture

The outgrowth method for isolation of primary cultured cells from resected PDACs has been previously described [19]. In brief, small tissue blocks (2-mm^3^) from freshly resected human PDAC tumors were minced and cultured on a 10 cm^2^ uncoated tissue culture plate in DMEM/F12+Glutamax (Gibco, Life Technologies), supplemented with 20% fetal bovine serum (FBS) (Gemini Bio-Products, West Sacramento, CA)+1x Penicillin-Streptomycin (Gibco). Primary cell lines are indicated as TAFs for tumor-associated fibroblasts isolated from a PDAC tumor sample or HPF for non-cancer-associated human pancreatic fibroblasts isolated from the pancreatic parenchyma remote from the tumor. Normal primary lung fibroblasts (LF) as a non-tumor/non-pancreas-associated fibroblast control have been previously characterized [20] and were maintained in DMEM (Gibco)+10% FBS+1x Pen-Strep. All primary cells characterized for miR-21 expression and used in co-culture experiments were maintained at low passage (p2–3).

Immortalized, non-transformed human pancreatic ductal epithelial cells (HPDE) were grown in keratinocyte serum-free media supplemented with epidermal growth factor and bovine pituitary extract (Gibco) [21]. Human pancreatic cancer cell lines BxPC-3 and MiaPaCa were obtained from the American Type Culture Collection (Rockville, MD) and maintained in DMEM+10% FBS+1x Pen-Strep.

### KRAS Sequencing

DNA was first extracted from FFPE samples with the QIAamp® DNA FFPE tissue kit (QIAGEN, Düsseldorf, Germany) or primary TAF cell lines via 0.2% SDS lysis buffer containing proteinase K, followed by 4°C isopropanol precipitation. PCR reactions were then performed (forward primer sequence –5′-GGCCTGCTGAAAATGACTGA-3′, reverse primer sequence 5′-GTCCTGCACCAGTAATATGC-3′) to amplify the *KRAS* exon 1 locus (as codon 12 is mutated in >80% of PDACs [22]) and then submitted to Laragen, Inc. Culver City, CA for sequencing on an ABI 3730XL Sequencer (Applied Biosystems, Life Technologies).

### qRT-PCR for microRNA-21

Utilizing the QIAGEN® system, microRNA was extracted from cells (miRNeasy Mini Kit), reverse transcribed (miScript® II Reverse Transcription Kit) and then qRT-PCR was performed (miScript® SYBR Green PCR Kit) with miScript® Primers for miR-21 and RNU6B as a housekeeping control.

### Overexpression/knockdown of Microrna-21

miScript® miR mimics and inhibitors were utilized for overexpression and knockdown experiments (QIAGEN). Cells were transfected with HiPerfect® Transfection reagent in the presence of miR-21 mimic or inhibitor. AllStars Negative Control siRNA or miScript® Inhibitor Negative Control were used as appropriate. The efficiency of miR-21 overexpression/knockdown experiments versus negative controls 48 hours after transfection is displayed in Figure S5.

### Co-culture Assays

HPFs were seeded at a density of 1×10^5^ in 6-well plates, and HPDE or MiaPaCa TCs were seeded at the same density in 6-well inserts with a 0.4 µM porous PET membrane (BD Biosciences, San Jose, CA). After 24 hours media was switched to serum-free DMEM/F12 and the inserts were placed in the wells. After 72 hours of co-culture, HPFs were collected for miR-21 qRT-PCR.

### Invasion Chamber Assays

Cells were first transfected with miR-21 mimic or inhibitor in serum-containing medium. After 24 hours, cells were washed with PBS and media was replaced. At 48 hours after transfection, cells were trypsinized, counted, and seeded with GFP-labeled MiaPaCa TC, which we have previously characterized [23], at a 1∶1 ratio and a density of 4×10^4^ cells per well in DMEM/F12+Glutamax+4% FBS. 24-well Matrigel™-coated invasion chambers with 8.0 µM pores (BD Biosciences) were first rehydrated then cells were added to the insert while media supplemented with 20% FBS was added to the bottom of the well to establish a serum gradient. After 24 hours of co-culture, GFP-positive TCs that had invaded through the membrane were counted in 5 evenly spaced non-overlapping visual fields at 10×magnification for each well. Each condition was performed in triplicate, and the entire experimental protocol was repeated ×2. Data shown is from one representative experiment.

### In Vivo Tumorigenesis Assays

Primary TAFs (3.5×10^5^) and BxPC-3 cells (3.5×10^5^) were injected orthotopically into NOD/SCID IL2Rγ null mice alone or in combination (n = 12, 4 mice in each group) as a 1∶1 Matrigel™:media suspension. 6 weeks following implantation, mice were sacrificed to assess for the presence of tumor, tumor size and weight.

### Statistical Analysis

Statistical analysis was performed with SPSS 20.0.0.1 (IBM, New York, NY). Patient survival was R-censored at 100 months and Kaplan-Meier analysis was informed by the log-rank test. Student t-test was used for comparison of means. Χ-square identified significant associations between miR-21 histoscores and clinicopathologic factors. Univariate Cox proportional hazard models were used to calculate hazard ratios for clinicopathologic factors with 95% CIs. A multivariate Cox regression analysis (MVA) was performed in a stepwise fashion with backward selection of statistically significant univariate parameters using P<0.10 as the initial entry criterion. Statistical significance was defined as P<0.05. Error bars ± SD.

## Results

### MicroRNA-21 Expression in the PDAC Stroma is Associated with Metastasis and Poor Prognosis

**Figure 1:**
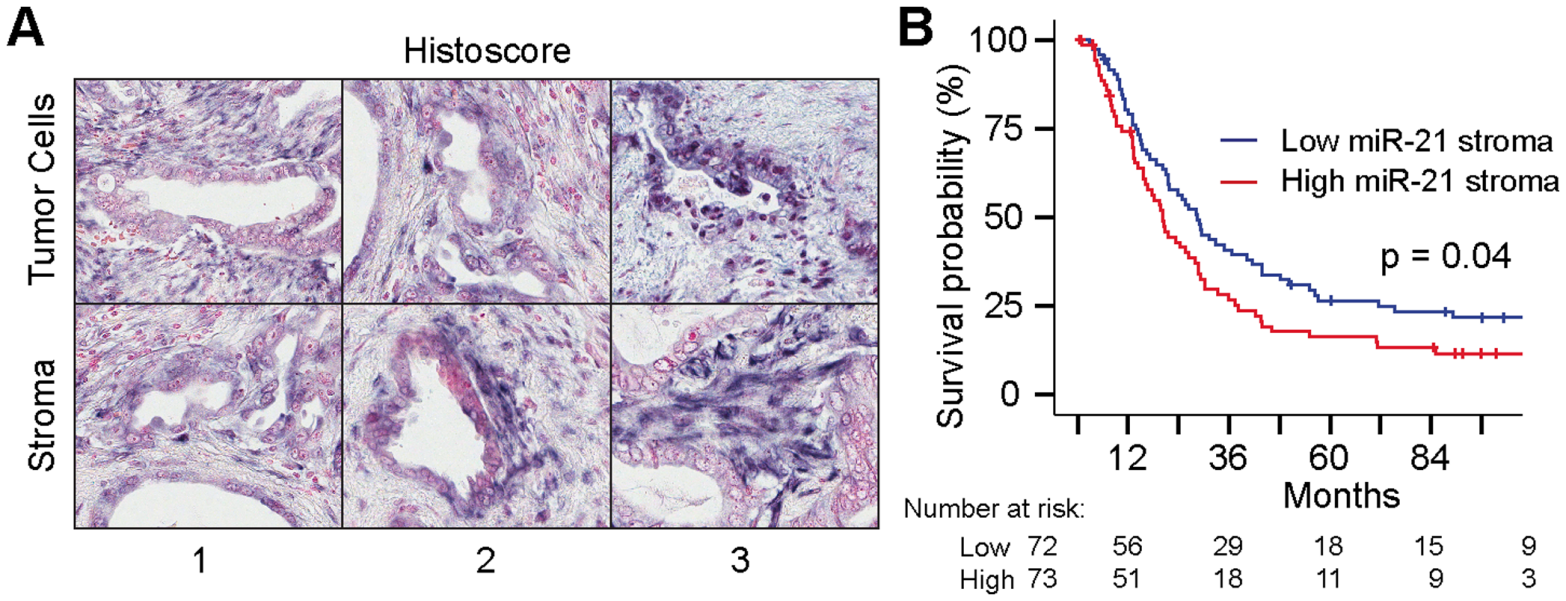
microRNA-21 expression in the PDAC stroma is associated with poor prognosis. (**A**) Representative images of histoscores for miR-21 *in situ* hybridization in PDAC tumor cells and stroma. These two cellular compartments were scored as 0 negative (not depicted), 1 weakly positive, 2 moderately positive, 3 strongly positive. (**B**) Kaplan-Meier analysis reveals that high miR-21 stromal expression is associated with decreased overall survival (P = 0.04). miR-21 expression intensity was dichotomized into high (n = 73) vs. low (n = 72) based on the median score of all tumors.

PDAC is associated with a dense stroma that contributes to tumorigenesis [24]. We hypothesized that miR-21 expression in the PDAC stroma correlated with clinical progression of disease. The UCLA PDAC TMA contains samples from 153 resected early-stage PDACs and was stained for miR-21 utilizing ISH. Representative images for histoscoring of TC and stroma is shown in Figure 1A. 78.4% of patients had a median histoscore for peritumoral stroma of ≥1 (Figure S1). Patients were dichotomized into miR-21 high (n = 73) or low (n = 72) based on the median histoscore = 1.5. On Kaplan-Meier survival analysis, high miR-21 stromal expression correlated with shorter overall survival (P = 0.04, Figure 1B), while miR-21 TC expression did not (Figure S1). As a means to explain the underlying mechanism of worse survival, miR-21 stromal expression was correlated with various histopathologic factors previously shown to be associated with prognosis – on both this TMA and an independent PDAC patient cohort [25] (Table 1). Interestingly, miR-21 in PDAC stroma did not correlate with tumor grade. It was strongly correlated with lymph node (LN) positivity (P = 0.004); 67% of miR-21 high patients had positive LNs while only 42% with low miR-21 had LN involvement. On Cox proportional hazards multivariate analysis, even after controlling for clinicopathologic variables associated with survival, stromal miR-21 expression on the TMA remained significant (HR = 1.56, P = 0.023) (Table 2). Taken together, these results reveal that miR-21 expression in the PDAC stroma is prognostically significant because it is correlated with TC invasion and metastasis.

**Table 1:** Correlation of miR-21 stromal expression level with clinicopathologic covariates.

		Low miR-21	High miR-21	P value
**Age**	**<65**	35 (45.5%)	29 (41.4%)	0.63
	**≥65**	42 (54.5%)	41 (58.6%)	
**Sex**	**Male**	40 (51.9%)	36 (51.4%)	0.95
	**Female**	37 (48.1%)	34 (48.6%)	
**AJCC Stage** a	**I**	27 (37.0%)	11 (15.7%)	
	**II**	45 (61.6%)	59 (84.3%)	0.008
	**IV**	1 (1.4%)	0 (0%)	
**Lymph Node**	**Negative**	42 (57.5%)	23 (33.3%)	0.004
	**Positive**	31 (42.5%)	46 (66.7%)	
**pTumor size^b^**	**T1**	13 (17.8%)	9 (12.9%)	
	**T2**	34 (46.6%)	26 (37.1%)	0.09
	**T3**	26 (35.6%)	35 (50.0%)	
**Grade**	**Low-mod**	44 (60.3%)	39 (55.7%)	0.58
	**High**	29 (39.7%)	31 (44.3%)	

a Pearson χ-square of miR-21 expression for stage I vs. II and b. T1+T2 vs. T3.

**Table 2:** Cox proportional hazard models for prognostic factors.

	Univariate analysis		Multivariate analysis	
	HR (95% CI)	P value	HR (95% CI)	P value
**Age (≥65/<65)**	1.2 (0.8–1.7)	0.30		
**Sex (female/male)**	1.5 (1.0–2.1)	0.04	1.5 (1.1–2.2)	0.03
**AJCC Stage (II**–**IV/I)**	1.6 (1.0–2.4)	0.04	–	
**Lymph node (pos/neg)**	1.8 (1.2–2.5)	0.003	1.6 (1.1–2.4)	0.01
**LVI (pos/neg)**	1.8 (1.0–3.3)	0.07		
**pT (pT3/pT1+pT2)**	1.0 (0.7–1.4)	0.93		
**Tumor size (>3 cm/≤3 cm)**	1.3 (0.9–1.9)	0.15		
**Grade (high/low)**	1.7 (1.2–2.4)	0.007	1.5 (1.0–2.2)	0.04
**Margin (R1/R0)** a	1.5 (0.9–2.5)	0.15		
**miR-21 stroma (high/low)**	1.5 (1.0–2.1)	0.04	1.6 (1.1–2.3)	0.02
**miR-21 tumor (high/low)**	1.1 (0.7–1.6)	0.70		

a Margins are classified as R1, macroscopically negative or R0, microscopically negative.

### MicroRNA-21 in the PDAC Stroma is Expressed in Activated Myofibroblasts

**Figure 2:**
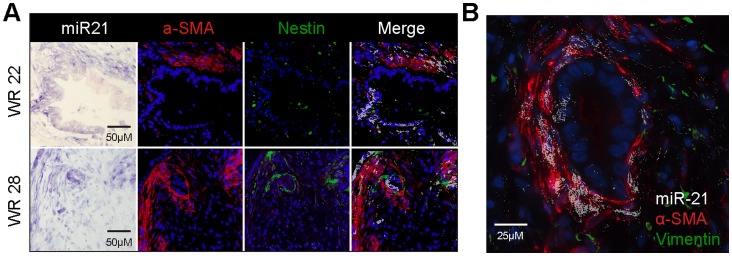
microRNA-21 in the PDAC stroma is expressed in activated myofibroblasts. (**A**) miR-21 *in situ* hybridization and co-immunofluorescence for α-SMA and nestin on serial sections of two human PDAC tumors (Whipple resection - WR 22 & 28) reveals miR-21 expression in a subset of activated myofibroblasts, not exclusive to stellate cells (nestin positive). miR-21 positive staining is white in the merge. (**B**) High-power magnification (40x) again reveals miR-21 expression in a subset of α-SMA expressing myofibroblasts. Vimentin, a marker for quiescent fibroblasts, does not localize with miR-21.

The PDAC stroma is comprised of a diverse cell population [24], including fibroblasts, activated myofibroblasts, stellate cells [26], inflammatory cells, and endothelial cells. Activated myofibroblasts and stellate cells are associated with PDAC TC invasion and chemotherapy resistance [4], [27]. Based on our previous results that stromal miR-21 expression is associated with prognosis, we next sought to determine the specific cell type expressing miR-21. An ISH-immunofluorescence digital overlay for miR-21 (white), the activated myofibroblast marker α-smooth muscle actin (red, α-SMA), and the stellate cell marker nestin (green) (Figure 2A) reveals that miR-21 is expressed in a subset of α-SMA and nestin positive cells. A larger magnification view (Figure 2B) of miR-21 (white), α-SMA (red), and the fibroblast marker vimentin (green) confirms these findings. These results reveal that miR-21 is expressed in a subpopulation of activated myofibroblasts and stellate cells, as opposed to simply representing a surrogate marker of these cell types.

### PDAC Tumor Cells Induce Tumor-associated Fibroblasts to Express MicroRNA-21

**Figure 3:**
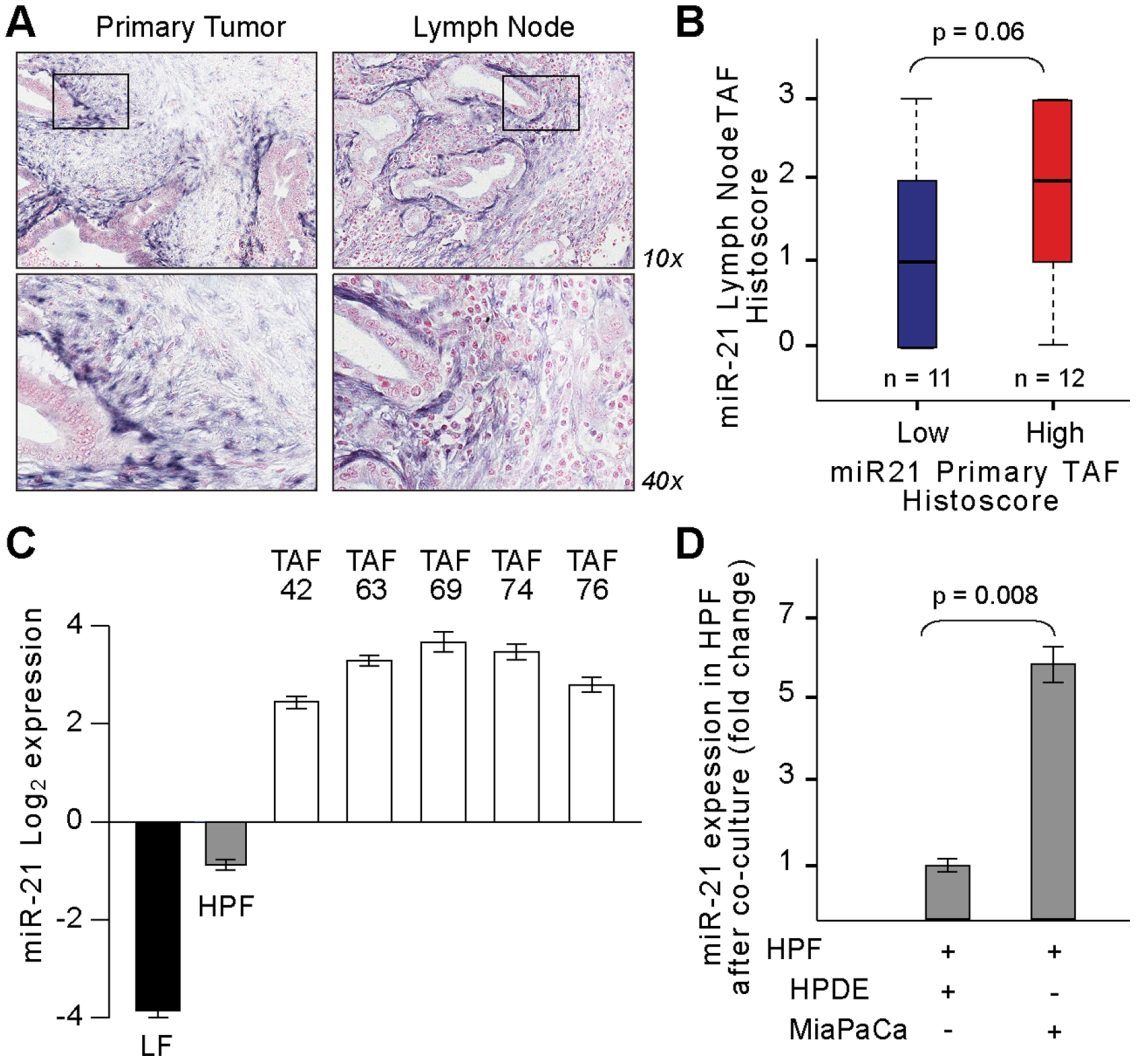
PDAC tumor cells induce tumor-associated fibroblasts (TAFs) to express microRNA-21. (**A**) Representative images of miR-21 *in situ* hybridization (ISH) on a patient-matched primary tumor and lymph node metastasis reveals high peritumoral expression of miR-21 in the stroma, decreasing in a radial gradient away from the tumor cells. (**B**) From miR-21 ISH on 23 patient-matched samples, miR-21 expression in the primary TAFs correlates with that of TAFs found in lymph node metastases (p = 0.06). miR-21 expression was dichotomized into high (n = 12) vs. low (n = 11) based on the median intensity level. (**C**) miR-21 expression in early-passage (p2) primary TAFs derived from resected human PDACs is elevated >8 times that of human pancreas fibroblasts (HPF) from noncancerous tissue. (**D**) Co-culture of HPFs with MiaPaCa tumor cells reveals a >5 fold increase in miR-21 in HPFs versus co-culture with normal human ductal epithelial (HPDE) cells. miR-21 expression was normalized to RNU6B. Error bars ± SD. Data are representative of 3 independent experiments.

PDAC TCs recruit supportive cells to their environment during tumor initiation and progression [27]. While the source of TAFs is unclear, it has been previously shown that TAFs do not possess genomic mutations but are activated through interactions with TCs [28]. We next sought to determine how miR-21 is expressed in TAFs and hypothesized that TCs induce them to upregulate the oncomir. To begin to answer this question, we identified and assembled 23 patient-matched cores of LN metastasis from TMA primary tumors. Figure 3A reveals that miR-21 expression in primary and LN TAFs is strongest in the region immediately surrounding the malignant ducts. The expression decreases along a radial gradient away from TCs. Strikingly, miR-21 expression in primary tumor TAFs showed a near significant correlation with miR-21 expression in TAFs from patient-matched LN metastases (P = 0.06, Figure 3B). Moreover, primary human PDAC TAFs isolated via the outgrowth method [19] express >8 fold higher miR-21 than noncancerous human fibroblasts isolated from a region of the pancreas remote from the cancer (Figure 3C). As more direct mechanistic evidence, miR-21 expression in normal HPFs was >5 fold higher when co-cultured with MiaPaCa TCs than when co-cultured with normal HPDE cells (Figure 3D). Validation that the primary TAFs are not derived from TCs included sequencing of *KRAS* and *in situ* immunofluorescence staining for specific epithelial and mesenchymal markers. These primary cell types are indeed *KRAS* wild-type, pan-cytokeratin negative, and express α-SMA, Vimentin, and GFAP (Figure S2–S3). To ensure that this primary culture did not include a contaminating population of tumor cells that had undergone epithelial-to-mesenchymal transition (EMT), we performed a tumorigenesis assay in an immunocompromised xenograft. At a count of 3.5×10^5^ cells, primary TAFs did not form tumors when injected in NOD/SCID IL2Rγ null mice (Figure S4). These results suggest that TCs could induce TAFs in both the primary tumor and LNs to express miR-21.

### microRNA-21 Expression in TAFs and Tumor Cells Drives Tumor Cell Invasion

**Figure 4:**
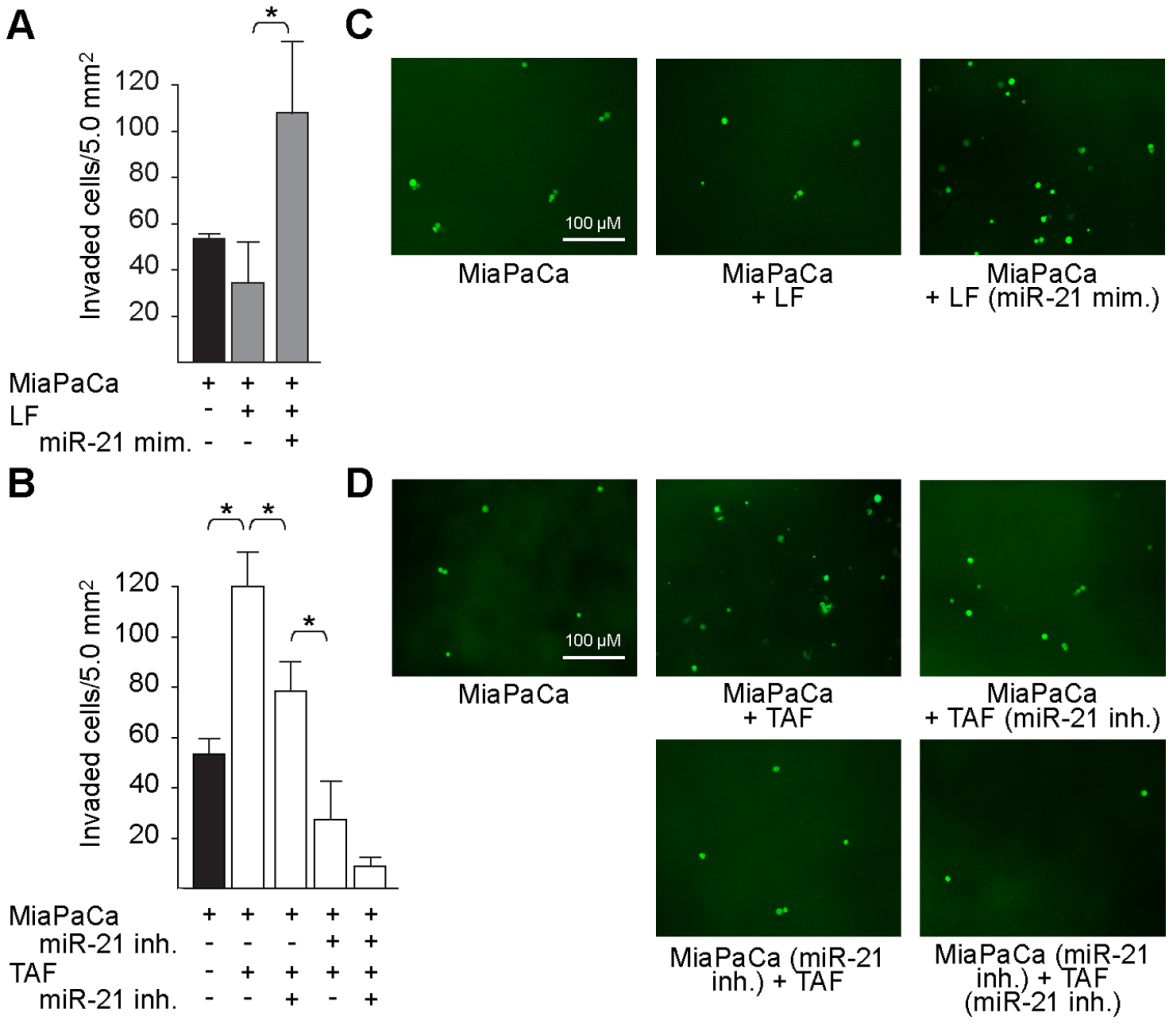
microRNA-21 expression in fibroblasts enhances tumor cell invasion and can be inhibited with an anti-miR. (**A**) Transfection of miR-21-low normal primary lung fibroblasts (LF) with miR-21 mimic enhances invasion of GFP-labeled MiaPaCa tumor cells (TCs) when co-cultured in modified Boyden chambers. *P<0.05. There was no difference in invasion in co-culture of LFs transfected with negative control versus TCs alone. Error bars ± SD. (B) Co-culture of TAFs with MiaPaCa TCs enhances invasion when compared to TCs alone. Transfection of miR-21-high TAFs with a miR-21 inhibitor decreases invasion of TCs. *P<0.05. Dual miR-21 inhibitor treatment of TCs and TAFs is the most effective at inhibiting TC invasion. Error bars ± SD. Data are representative of three independent experiments. (**C–D**) Representative photomicrographs of GFP-labeled TCs that had invaded through the membrane for each treatment condition.

Based on the correlative data between miR-21 expression in TAFs and LN involvement on the UCLA TMA, we hypothesized that miR-21 expression in PDAC TAFs increases invasiveness of PDAC TCs. MiaPaCa TCs were selected for the modified Boyden chamber experiments as they have been previously shown to express high miR-21 levels [12]. Overexpression experiments were carried out utilizing miR-21 mimics in early-passage normal LFs with low baseline miR-21 expression (Figure 3C). When TCs were co-cultured with miR-21 overexpressing LFs, TC invasion was significantly increased compared to TCs+negative control-treated LFs (P = 0.05, Figure 4A). In fact, co-culture of TCs+low miR-21 LFs did not increase invasion as compared to TCs alone (Figure 4A). To determine the effect of miR-21 inhibition on TC invasion, we then tested TCs co-cultured with primary human early-passage PDAC TAFs that express high levels of miR-21 (Figure 3C). Co-culture of TAFs with TCs significantly increased the number of cells invaded as compared to TCs alone (P = 0.01, Figure 4B). This increase was significantly abrogated by inhibition of miR-21 in TAFs or TCs (P = 0.04 and P<0.001 respectively). Strikingly, inhibition of miR-21 in both TCs and TAFs was additive and resulted in the greatest inhibition of TC invasion. These results suggest that miR-21 may be a promising dual TC and TAF target in human PDAC to decrease invasion and metastasis of TCs.

## Discussion

Pancreatic cancer has the highest stromal volume of all solid tumors [24], and strategies to reduce the pro-tumorigenic stroma may improve delivery of chemotherapy drugs to TCs and increase treatment efficacy in this fatal disease [29], [30]. We investigated the hypothesis that miR-21 expression in the PDAC stroma increases invasion and metastasis of TCs. We found that miR-21 in activated peritumoral myofibroblasts is associated with LN metastasis and shorter survival. Our results also suggest that the TCs induce the fibroblasts to express miR-21. Importantly, inhibition of miR-21 in PDAC TAFs decreases TC invasion.

In multiple independent studies, high miR-21 expression in human PDAC TCs is correlated with shorter survival [11], [13], [14]. As an explanation of this prognostic significance, *in vitro* modulation of miR-21 in PDAC TCs increases proliferation, invasion, and gemcitabine chemoresistance [12], [13]. However, miR-21 expression in the non-TC compartment plays a role in the histopathologic progression of cancer and non-cancerous conditions. *In situ* hybridization of 130 colon and 67 rectal cancer specimens revealed that miR-21 expression in the stroma predicts shorter disease-free survival of stage II patients [16], [31]. In the lung, miR-21 expression is increased in myofibroblasts of patients with pulmonary fibrosis as compared to healthy lung tissue [32]. Its increase is driven by TGF-β released from the epithelial cells, and it potentiates the development of worsening scar tissue. In the heart, miR-21 expression increases in cardiac fibroblasts of the failing heart [33]. In an *in vivo* pressure-overload-induced disease model, inhibition of miR-21 using an antagomiR decreased interstitial fibrosis and cardiac hypertrophy. In the kidney, miR-21 expression increases fibrosis due to ureteral obstruction in a murine model [34]. Inhibition of miR-21 *in vivo* significantly attenuated fibrosis development. The architecture of each of these three non-cancerous conditions resembles that of the PDAC stromal environment. Furthermore, in regards to drug delivery, miR-21 in the stromal environment has been shown to decrease angiogenesis by inhibiting RhoB in endothelial cells [35]. These mechanisms are likely present in the majority of patients with PDAC as we show miR-21 expression in TAFs of 78.4% of early-stage PDACs.

In our previous analysis, we identified a prognostic gene signature of 171 genes in PDAC TCs [7]. However, genes with prognostic significance are not just limited to the TC compartment but have also been identified in the stroma of other malignancies. In breast cancer, 53 laser capture microdissected human samples were used to identify gene expression changes that clustered to angiogenesis, immune and hypoxic responses. These stromal changes stratified survival outcome for multiple subtypes of breast cancer and were independent of standard clinicopathologic factors [36]. Combining the stromal gene expression changes with standard prognostic factors enhanced the survival prediction accuracy in independent datasets. Likewise, in non-small cell lung cancer, using 15 patient-matched cancer-associated and normal fibroblasts, 46 differentially expressed genes were identified [37]. A subset of 11 of these genes formed a prognostic signature and were associated with survival in independent patient cohorts. While a similar analysis has not been done for PDAC, these studies provide proof of principle that stromal gene expression changes are important for biologic progression of disease. We identify that stromal miR-21 expression in PDAC is associated with disease progression as manifested by LN metastasis, poor prognosis and TC invasion.

During PDAC tumor development – from low to high grade PanINs to invasive cancer – the stromal volume expands. The predominant components of this rich microenvironment include fibroblasts as well as stellate, inflammatory and endothelial cells. The fibroblasts and stellate cells are responsible for production of the dense fibrotic matrix [19]. They are recruited to the PDAC tumor microenvironment by paracrine mediators secreted by TCs. These mediators include TGFβ, PDGF, and VEGF [27]. After recruitment, stellate cells are activated and fibroblasts transform from a quiescent to a myofibroblast-like state via growth factors or cytokines released from TCs, or the hypoxic environment of PDAC [19], [38], [39]. Interestingly, the genomic profile of activated and quiescent stellate cells is identical, suggesting that the gene expression differences between the two states are mediated by cues from the environment or epigenetic and post-translational modifications [28]. Once activated, they enhance PDAC progression and have been shown to contribute to chemoresistance, tumor growth, and metastasis in cell culture and *in vivo*. Therefore, we investigated the etiology of increased miR-21 expression in the PDAC stroma and focused on hypoxia and induction by the TCs. miR-21 did not increase in primary TAFs grown under hypoxic conditions (data not shown). Rather, we found that miR-21 expression was highest around TCs and decreased in a radial gradient away from malignant ducts. Assuming that the O_2_ levels are constant in these two regions, it is suggestive that, much like TAF activation, TCs induce TAFs to increase expression of miR-21. We also observed a near-significant correlation for miR-21 expression between primary tumor TAFs and TAFs found in LN metastases. While recent studies have characterized the invasive potential of fibroblasts themselves [40], providing a possible explanation for how miR-21-high expressing TAFs in the primary tumor could directly invade to the LN, we have provided evidence that miR-21 expression can be induced in non-cancer associated fibroblasts by TCs. That the highest intensity of miR-21 TAF expression is universally peritumoral and that we do not observe isolated islands of miR-21-high expressing TAFs is further evidence of an induction mechanism.

miRs, and miR-21 in particular, function by regulating a network of genes [5]. However, to our knowledge, the miR-21 “targetome” has not been identified in PDAC TAFs or TAFs of other solid cancers. As potential mechanisms of enhanced TC invasion, miR-21 has been previously shown to promote matrix remodeling and regulate TGF-β-induced epithelial-to-mesenchymal transition [41]. We tested several markers of EMT (*SNAI1, ZEB1, S100A4, CDH1/2, VIM*) in TCs and matrix remodeling (*TIMP2, RECK, MMP2/9*) in LFs with miR-21 overexpression and TAFs with miR-21 inhibition, and did not identify changes in transcript levels via qRT-PCR (data not shown). A potential explanation for these findings is that miRs inhibit translation of target genes by (i) transcript degradation if the binding affinity is high with near-perfect complementary base-pairing or (ii) cessation of translation through the RISC complex without degradation of mRNA for less specific binding [42]. Therefore, the lack of quantitative changes in these transcripts does not exclude them as miR-21 targets in PDAC TAFs. The next step is CLIP-sequencing, the most accurate method to identify miR “targetomes,” whereby mRNA and miR pairs bound to Argonaute protein are sequenced [43], [44].

miRs have the potential to be effective therapeutic targets. Silencing of miRs using intravenously administered chemically engineered oligonucleotides (a.k.a. “antagomiRs”) has been successfully completed in many solid organs, *in vivo* models, and disease states [45], [46]. For example, miR-122 expression in the liver is associated with cholesterol biosynthesis and hepatitis C virus (HCV) propagation. Systemically administered LNA antagomiRs to miR-122 in nonhuman primates successfully reduced plasma cholesterol [47], and HCV viremia and associated liver changes [48]. A phase I/II human clinical trial using anti-miR-122 for patients with hypercholesterolemia is now in progress (www.santaris.org). To our knowledge, effective distribution of LNA antagomiRs via systemic administration to the pancreas and hypovascular PDACs has not been demonstrated. This will be a challenge to accomplish before antagomiR therapy is possible for this disease.

## Conclusions

Our results reveal that miR-21 expression in PDAC activated myofibroblasts is associated with poor prognosis, LN metastases, and TC invasion. miR-21 inhibition in TCs and TAFs significantly decreases TC invasion in cell culture. These results, taken in conjunction with previous miR-21 findings on PDAC TC chemoresistance, suggest that miR-21 is a promising dual TC and TAF target in PDAC.

## Supporting Information

Figure S1 **microRNA-21 staining in tumor cells was not correlated with survival.** (**A**) High miR-21 staining in the tumor cells did not correlate with worse survival. (**B–C**) Distribution of histoscores for tumor-associated fibroblasts (TAF) and tumor cells reveals that ≈80% of early stage PDAC tumors express miR-21 in the stroma.
(TIF)

Figure S2 **Tumor-associated fibroblasts (TAFs) are** ***KRAS*** **wild type.** Primary TAFs isolated via the outgrowth method were sequenced for *KRAS* mutation at codon 12 and 13. All primary TAFs were identified as *KRAS* wild type. Patient-matched FFPE tumor samples were also sequenced and all returned positive for *KRAS* mutation at codon 12 (data not shown). As a positive control, the pancreatic cancer cell line L3.6pl harbors the G12D mutation. This provides strong evidence that these primary TAFs are not tumor-cell derived.
(TIF)

Figure S3 **Primary human tumor-associated fibroblasts (TAFs) show an activated myofibroblast phenotype.** *In situ* immunofluorescence staining of primary TAFs derived from PDAC human tumor samples and human pancreatic ductal epithelial (HPDE) cells as a control for α-smooth muscle actin (α-SMA), Vimentin, glial fibrillary acid protein (GFAP), and pan-cytokeratin (PanCK). These representative stains for TAF cell lines reveal them to be spindle-shaped, positive for vimentin and weakly positive for GFAP, consistent with a fibroblast phenotype that has become activated in culture (positive α-SMA). All are negative for the epithelial marker PanCK.
(TIF)

Figure S4 **Primary human tumor-associated fibroblasts (TAFs) enhance tumor growth but do not form** ***de novo*** **tumors.** Primary pancreatic TAFs were orthotopically injected in NOD/SCID IL2Rγ null mice (3.5×10^5^ cells) with or without BxPC-3 tumor cells (1∶1 ratio). Necropsy at 6 weeks revealed that coinjection of TAFs with tumor cells enhances tumor growth but does not produce a tumor when injected alone. Representative photographs of pancreas (white outline) and tumor (yellow dotted line).
(TIF)

Figure S5 ***In vitro*** **microRNA-21 overexpression/knockdown.** Transfection with miR-21 mimic produces overexpression in normal lung fibroblasts (low baseline miR-21 expression) and anti-sense miR-21 leads to knockdown in primary tumor-associated fibroblast cell lines (TAF) as assessed by qRT-PCR.
(TIF)

Table S1 **TMA baseline patient characteristics.** Abbreviations: TMA, tissue microarray, LVI, lymphovascular invasion.
(DOCX)

## References

[1] American Cancer Society (2013) Cancer facts & figures 2013. Atlanta, GA: 1–64. Available: http://www.cancer.org/research/cancerfactsfigures/cancerfactsfigures/cancer-facts-figures-2013. Accessed 2013 Jul 29.

[2] Martisian LM, Aizenberg R, Rosenzweig A (2012) The Alarming Rise of Pancreatic Cancer Deaths in the United States: Why We Need to Stem the Tide Today. Pancreatic Cancer Action Network: 1–12. Available: http://www.pancan.org/section_research/reports/pdf/incidence_report_2012.pdf. Accessed 2013 Jul 29.

[3] Iacobuzio-Donahue CA, Fu B, Yachida S, Luo M, Abe H, et al (2009) DPC4 Gene Status of the Primary Carcinoma Correlates With Patterns of Failure in Patients With Pancreatic Cancer. Journal of Clinical Oncology 27: 1806–1813 doi:10.1200/JCO.2008.17.7188 19273710 10.1200/JCO.2008.17.7188 PMC2668706

[4] Hwang RF, Moore T, Arumugam T, Ramachandran V, Amos KD, et al (2008) Cancer-associated stromal fibroblasts promote pancreatic tumor progression. Cancer Research 68: 918–926 doi:10.1158/0008-5472.CAN-07-5714 18245495 10.1158/0008-5472.CAN-07-5714 PMC2519173

[5] Esquela-Kerscher A, Slack FJ (2006) Oncomirs - microRNAs with a role in cancer. Nat Rev Cancer 6: 259–269 doi:10.1038/nrc1840 16557279 10.1038/nrc1840

[6] Lu J, Getz G, Miska EA, Alvarez-Saavedra E, Lamb J, et al (2005) MicroRNA expression profiles classify human cancers. Nature 435: 834–838 doi:10.1038/nature03702 15944708 10.1038/nature03702

[7] Donahue TR, Tran LM, Hill R, Li Y, Kovochich A, et al (2012) Integrative survival-based molecular profiling of human pancreatic cancer. Clin Cancer Res 18: 1352–1363 doi:10.1158/1078-0432.CCR-11-1539 22261810 10.1158/1078-0432.CCR-11-1539 PMC3816537

[8] Meng F, Henson R, Wehbe Janek H, Ghoshal K, Jacob ST, et al (2007) MicroRNA-21 regulates expression of the PTEN tumor suppressor gene in human hepatocellular cancer. YGAST 133: 647–658 doi:10.1053/j.gastro.2007.05.022 10.1053/j.gastro.2007.05.022 PMC4285346 17681183

[9] Asangani IA, Rasheed SAK, Nikolova DA, Leupold JH, Colburn NH, et al (2008) MicroRNA-21 (miR-21) post-transcriptionally downregulates tumor suppressor Pdcd4 and stimulates invasion, intravasation and metastasis in colorectal cancer. Oncogene 27: 2128–2136 doi:10.1038/sj.onc.1210856 17968323 10.1038/sj.onc.1210856

[10] Bloomston M, Frankel WL, Petrocca F, Volinia S, Alder H, et al (2007) MicroRNA expression patterns to differentiate pancreatic adenocarcinoma from normal pancreas and chronic pancreatitis. JAMA 297: 1901–1908 doi:10.1001/jama.297.17.1901 17473300 10.1001/jama.297.17.1901

[11] Dillhoff M, Liu J, Frankel W, Croce C, Bloomston M (2008) MicroRNA-21 is overexpressed in pancreatic cancer and a potential predictor of survival. J Gastrointest Surg 12: 2171–2176 doi:10.1007/s11605-008-0584-x 18642050 10.1007/s11605-008-0584-x PMC4055565

[12] Moriyama T, Ohuchida K, Mizumoto K, Yu J, Sato N, et al (2009) MicroRNA-21 modulates biological functions of pancreatic cancer cells including their proliferation, invasion, and chemoresistance. Molecular Cancer Therapeutics 8: 1067–1074 doi:10.1158/1535-7163.MCT-08-0592 19435867 10.1158/1535-7163.MCT-08-0592

[13] Giovannetti E, Funel N, Peters GJ, Del Chiaro M, Erozenci LA, et al (2010) MicroRNA-21 in pancreatic cancer: correlation with clinical outcome and pharmacologic aspects underlying its role in the modulation of gemcitabine activity. Cancer Research 70: 4528–4538 doi:10.1158/0008-5472.CAN-09-4467 20460539 10.1158/0008-5472.CAN-09-4467

[14] Hwang JH, Voortman J, Giovannetti E, Steinberg SM, Leon LG, et al (2010) Identification of microRNA-21 as a biomarker for chemoresistance and clinical outcome following adjuvant therapy in resectable pancreatic cancer. PLoS ONE 5: e10630 doi:10.1371/journal.pone.0010630.t009 20498843 10.1371/journal.pone.0010630 PMC2871055

[15] Rieu du MC, Torrisani J, Selves J, Saati Al T, Souque A, et al (2010) MicroRNA-21 is induced early in pancreatic ductal adenocarcinoma precursor lesions. Clin Chem 56: 603–612 doi:10.1373/clinchem.2009.137364 20093556 10.1373/clinchem.2009.137364

[16] Kjaer-Frifeldt S, Hansen TF, Nielsen BS, Joergensen S, Lindebjerg J, et al (2012) The prognostic importance of miR-21 in stage II colon cancer: a population-based study. Br J Cancer 107: 1169–1174 doi:10.1038/bjc.2012.365 23011541 10.1038/bjc.2012.365 PMC3461159

[17] Nielsen BS, Jørgensen S, Fog JU, Søkilde R, Christensen IJ, et al (2011) High levels of microRNA-21 in the stroma of colorectal cancers predict short disease-free survival in stage II colon cancer patients. Clin Exp Metastasis 28: 27–38 doi:–10.1007/s10585-010-9355–7 21069438 10.1007/s10585-010-9355-7 PMC2998639

[18] Manuyakorn A, Paulus R, Farrell JJ, Dawson NA, Tze S, et al (2010) Cellular histone modification patterns predict prognosis and treatment response in resectable pancreatic adenocarcinoma: results from RTOG 9704. Journal of Clinical Oncology 28: 1358–1365 doi:10.1200/JCO.2009.24.5639 20142597 10.1200/JCO.2009.24.5639 PMC2834495

[19] Bachem MG, Schünemann M, Ramadani M, Siech M, Beger H, et al (2005) Pancreatic carcinoma cells induce fibrosis by stimulating proliferation and matrix synthesis of stellate cells. YGAST 128: 907–921 doi:10.1053/j.gastro.2004.12.036 10.1053/j.gastro.2004.12.036 15825074

[20] Huang M, Sharma S, Zhu LX, Keane MP, Luo J, et al (2002) IL-7 inhibits fibroblast TGF-beta production and signaling in pulmonary fibrosis. J Clin Invest 109: 931–937 doi:10.1172/JCI14685 11927620 10.1172/JCI14685 PMC150933

[21] Liu N, Furukawa T, Kobari M, Tsao MS (1998) Comparative phenotypic studies of duct epithelial cell lines derived from normal human pancreas and pancreatic carcinoma. The American Journal of Pathology 153: 263–269 doi:10.1016/S0002-9440(10)65567-8 9665487 10.1016/S0002-9440(10)65567-8 PMC1852927

[22] Laghi L, Orbetegli O, Bianchi P, Zerbi A, Di Carlo V, et al (2002) Common occurrence of multiple K-RAS mutations in pancreatic cancers with associated precursor lesions and in biliary cancers. Oncogene 21: 4301–4306 doi:10.1038/sj.onc.1205533 12082617 10.1038/sj.onc.1205533

[23] Nguyen Kovochich A, Arensman M, Lay AR, Rao NP, Donahue TR, et al (2013) HOXB7 promotes invasion and predicts survival in pancreatic adenocarcinoma. Cancer 119: 529–539 doi:10.1002/cncr.27725 22914903 10.1002/cncr.27725 PMC3867310

[24] Neesse A, Michl P, Frese KK, Feig C, Cook N, et al (2011) Stromal biology and therapy in pancreatic cancer. Gut 60: 861–868 doi:10.1136/gut.2010.226092 20966025 10.1136/gut.2010.226092

[25] Cameron JL, Riall TS, Coleman J, Belcher KA (2006) One thousand consecutive pancreaticoduodenectomies. Annals of Surgery 244: 10–15 doi:10.1097/01.sla.0000217673.04165.ea 16794383 10.1097/01.sla.0000217673.04165.ea PMC1570590

[26] Omary MB, Lugea A, Lowe AW, Pandol SJ (2007) The pancreatic stellate cell: a star on the rise in pancreatic diseases. J Clin Invest 117: 50–59 doi:10.1172/JCI30082 17200706 10.1172/JCI30082 PMC1716214

[27] Vonlaufen A, Joshi S, Qu C, Phillips PA, Xu Z, et al (2008) Pancreatic stellate cells: partners in crime with pancreatic cancer cells. Cancer Research 68: 2085–2093 doi:10.1158/0008-5472.CAN-07-2477 18381413 10.1158/0008-5472.CAN-07-2477

[28] Campbell I, Qiu W, Haviv I (2011) Genetic changes in tumour microenvironments. J Pathol 223: 450–458 doi:10.1002/path.2842 21294119 10.1002/path.2842

[29] Olive KP, Jacobetz MA, Davidson CJ, Gopinathan A, McIntyre D, et al (2009) Inhibition of Hedgehog signaling enhances delivery of chemotherapy in a mouse model of pancreatic cancer. Science 324: 1457–1461 doi:10.1126/science.1171362 19460966 10.1126/science.1171362 PMC2998180

[30] Hoff Von DD, Ramanathan RK, Borad MJ, Laheru DA, Smith LS, et al (2011) Gemcitabine plus nab-paclitaxel is an active regimen in patients with advanced pancreatic cancer: a phase I/II trial. Journal of Clinical Oncology 29: 4548–4554 doi:10.1200/JCO.2011.36.5742 21969517 10.1200/JCO.2011.36.5742 PMC3565012

[31] Nelson PT, Baldwin DA, Scearce LM, Oberholtzer JC, Tobias JW, et al (2004) Microarray-based, high-throughput gene expression profiling of microRNAs. Nat Methods 1: 155–161 doi:10.1038/nmeth717 15782179 10.1038/nmeth717

[32] Liu G, Friggeri A, Yang Y, Milosevic J, Ding Q, et al (2010) miR-21 mediates fibrogenic activation of pulmonary fibroblasts and lung fibrosis. J Exp Med 207: 1589–1597 doi:10.1084/jem.20100035 20643828 10.1084/jem.20100035 PMC2916139

[33] Thum T, Gross C, Fiedler J, Fischer T, Kissler S, et al (2008) MicroRNA-21 contributes to myocardial disease by stimulating MAP kinase signalling in fibroblasts. Nature 456: 980–984 doi:10.1038/nature07511 19043405 10.1038/nature07511

[34] Chau BN, Xin C, Hartner J, Ren S, Castano AP, et al (2012) MicroRNA-21 promotes fibrosis of the kidney by silencing metabolic pathways. Sci Transl Med 4: 121ra18 doi:10.1126/scitranslmed.3003205 10.1126/scitranslmed.3003205 PMC3672221 22344686

[35] Sabatel C, Malvaux L, Bovy N, Deroanne C, Lambert V, et al (2011) MicroRNA-21 exhibits antiangiogenic function by targeting RhoB expression in endothelial cells. PLoS ONE 6: e16979 doi:10.1371/journal.pone.0016979.g006 21347332 10.1371/journal.pone.0016979 PMC3037403

[36] Finak G, Bertos N, Pepin F, Sadekova S, Souleimanova M, et al (2008) Stromal gene expression predicts clinical outcome in breast cancer. Nature Medicine 14: 518–527 doi:10.1038/nm1764 10.1038/nm1764 18438415

[37] Navab R, Strumpf D, Bandarchi B, Zhu C-Q, Pintilie M, et al (2011) Prognostic gene-expression signature of carcinoma-associated fibroblasts in non-small cell lung cancer. P Natl Acad Sci Usa 108: 7160–7165 doi:−10.1073/pnas.1014506108/−/DCSupplemental/st01.doc 10.1073/pnas.1014506108 PMC3084093 21474781

[38] Bachem MG, Schneider E, Gross H, Weidenbach H, Schmid RM, et al (1998) Identification, culture, and characterization of pancreatic stellate cells in rats and humans. YGAST 115: 421–432. 10.1016/s0016-5085(98)70209-4 9679048

[39] Apte MV, Haber PS, Darby SJ, Rodgers SC, McCaughan GW, et al (1999) Pancreatic stellate cells are activated by proinflammatory cytokines: implications for pancreatic fibrogenesis. Gut 44: 534–541. 10075961 10.1136/gut.44.4.534 PMC1727467

[40] Brentnall TA, Lai LA, Coleman J, Bronner MP, Pan S, et al (2012) Arousal of Cancer-Associated Stroma: Overexpression of Palladin Activates Fibroblasts to Promote Tumor Invasion. PLoS ONE 7: e30219 doi:10.1371/journal.pone.0030219.s004 22291919 10.1371/journal.pone.0030219 PMC3264580

[41] Brønnum H, Andersen DC, Schneider M, Sandberg MB, Eskildsen T, et al (2013) miR-21 Promotes Fibrogenic Epithelial-to-Mesenchymal Transition of Epicardial Mesothelial Cells Involving Programmed Cell Death 4 and Sprouty-1. PLoS ONE 8: e56280 doi:10.1371/journal.pone.0056280.s011 23441172 10.1371/journal.pone.0056280 PMC3575372

[42] Guo H, Ingolia NT, Weissman JS, Bartel DP (2010) Mammalian microRNAs predominantly act to decrease target mRNA levels. Nature 466: 835–840 doi:10.1038/nature09267 20703300 10.1038/nature09267 PMC2990499

[43] Chi SW, Zang JB, Mele A, Darnell RB (2009) Argonaute HITS-CLIP decodes microRNA-mRNA interaction maps. Nature 460: 479–486 doi:10.1038/nature08170 19536157 10.1038/nature08170 PMC2733940

[44] Thomson DW, Bracken CP, Goodall GJ (2011) Experimental strategies for microRNA target identification. Nucleic Acids Res 39: 6845–6853 doi:10.1093/nar/gkr330 21652644 10.1093/nar/gkr330 PMC3167600

[45] Krützfeldt J, Rajewsky N, Braich R, Rajeev KG, Tuschl T, et al (2005) Silencing of microRNAs in vivo with ‘antagomirs’. Nature 438: 685–689 doi:10.1038/nature04303 16258535 10.1038/nature04303

[46] van Rooij E, Purcell AL, Levin AA (2012) Developing microRNA therapeutics. Circ Res 110: 496–507 doi:10.1161/CIRCRESAHA.111.247916 22302756 10.1161/CIRCRESAHA.111.247916

[47] Elmén J, Lindow M, Schütz S, Lawrence M, Petri A, et al (2008) LNA-mediated microRNA silencing in non-human primates. Nature 452: 896–899 doi:10.1038/nature06783 18368051 10.1038/nature06783

[48] Lanford RE, Hildebrandt-Eriksen ES, Petri A, Persson R, Lindow M, et al (2010) Therapeutic silencing of microRNA-122 in primates with chronic hepatitis C virus infection. Science 327: 198–201 doi:10.1126/science.1178178 19965718 10.1126/science.1178178 PMC3436126

