# Supplementary figures and images for: MicroRNA-21 in Pancreatic Ductal Adenocarcinoma Tumor-Associated Fibroblasts Promotes Metastasis
Source: PLoS One. 2013 Aug 22;8(8):e71978. doi: 10.1371/journal.pone.0071978 (PMC3750050; doi:10.1371/journal.pone.0071978)

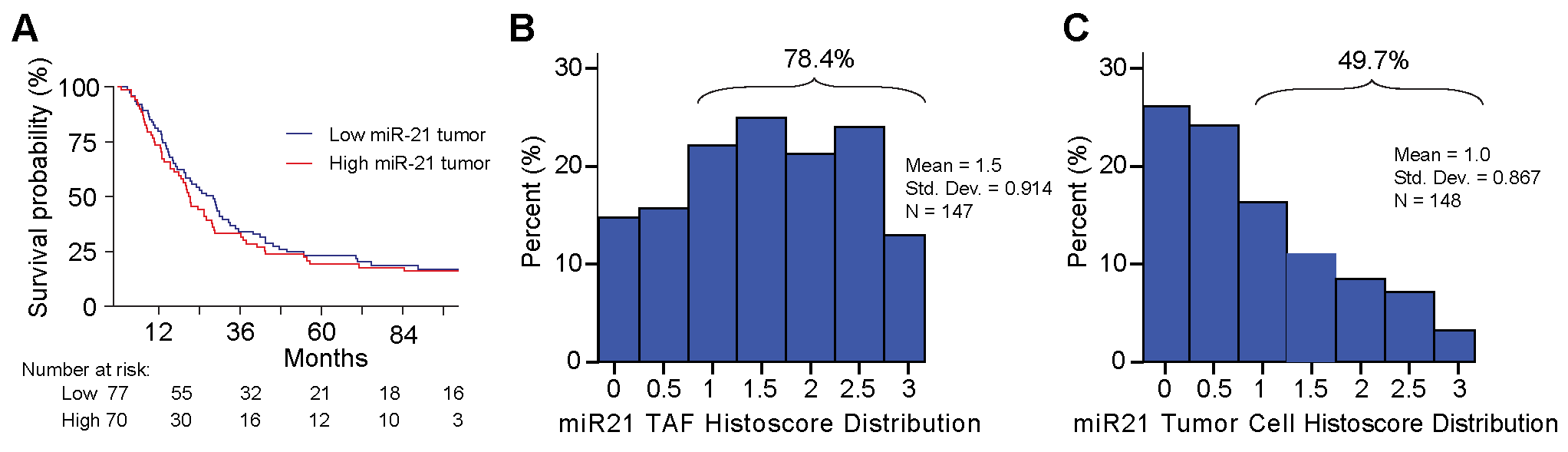

Supplement: Figure S1 — microRNA-21 staining in tumor cells was not correlated with survival. (A) High miR-21 staining in the tumor cells did not correlate with worse survival. (B–C) Distribution of histoscores for tumor-associated fibroblasts (TAF) and tumor cells reveals that ≈80% of early stage PDAC tumors express miR-21 in the stroma. (TIF) [file pone.0071978.s001.tif]

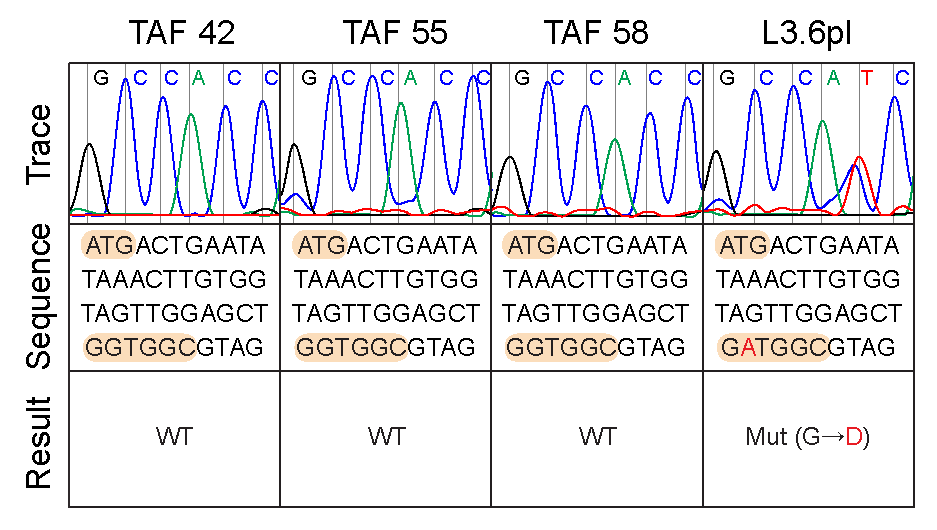

Supplement: Figure S2 — Tumor-associated fibroblasts (TAFs) are KRAS wild type. Primary TAFs isolated via the outgrowth method were sequenced for KRAS mutation at codon 12 and 13. All primary TAFs were identified as KRAS wild type. Patient-matched FFPE tumor samples were also sequenced and all returned positive for KRAS mutation at codon 12 (data not shown). As a positive control, the pancreatic cancer cell line L3.6pl harbors the G12D mutation. This provides strong evidence that these primary TAFs are not tumor-cell derived. (TIF) [file pone.0071978.s002.tif]

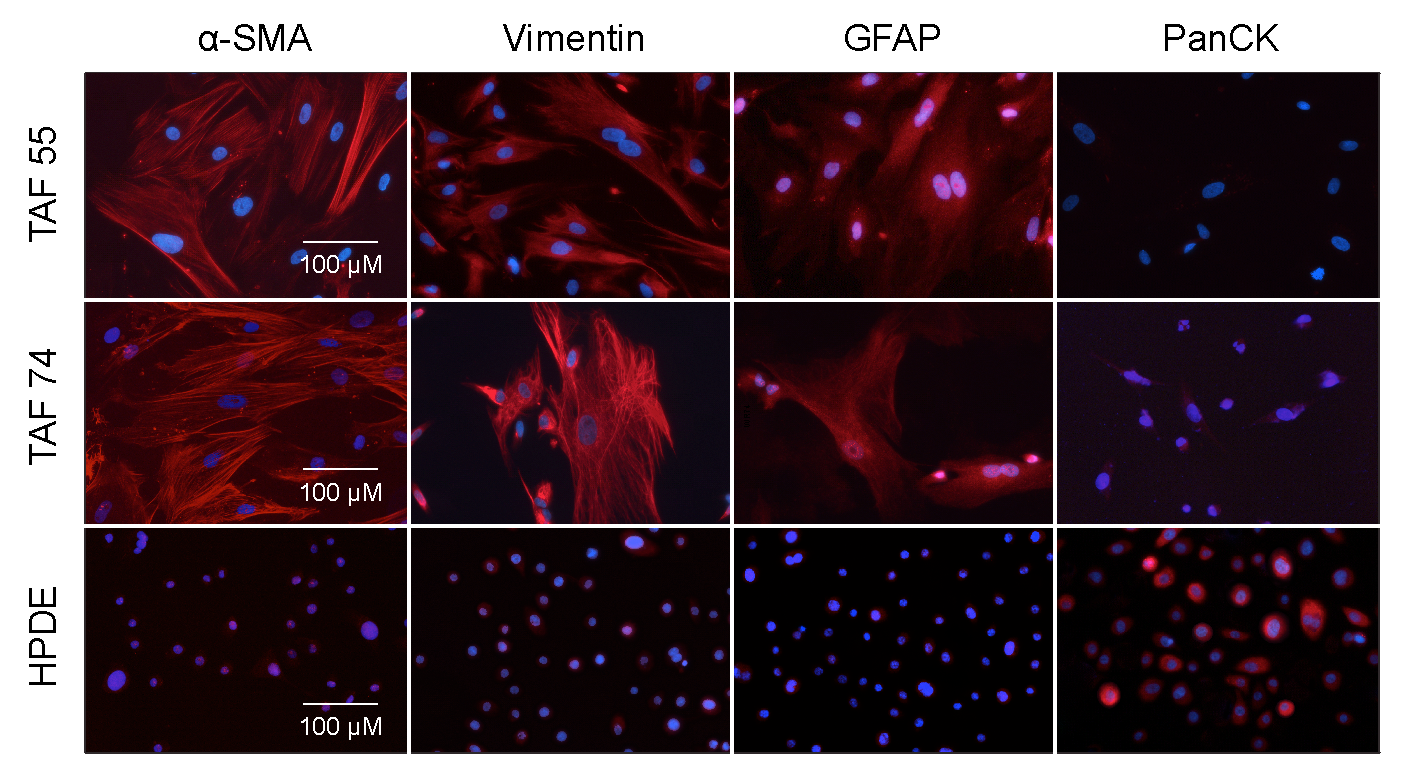

Supplement: Figure S3 — Primary human tumor-associated fibroblasts (TAFs) show an activated myofibroblast phenotype. In situ immunofluorescence staining of primary TAFs derived from PDAC human tumor samples and human pancreatic ductal epithelial (HPDE) cells as a control for α-smooth muscle actin (α-SMA), Vimentin, glial fibrillary acid protein (GFAP), and pan-cytokeratin (PanCK). These representative stains for TAF cell lines reveal them to be spindle-shaped, positive for vimentin and weakly positive for GFAP, consistent with a fibroblast phenotype that has become activated in culture (positive α-SMA). All are negative for the epithelial marker PanCK. (TIF) [file pone.0071978.s003.tif]

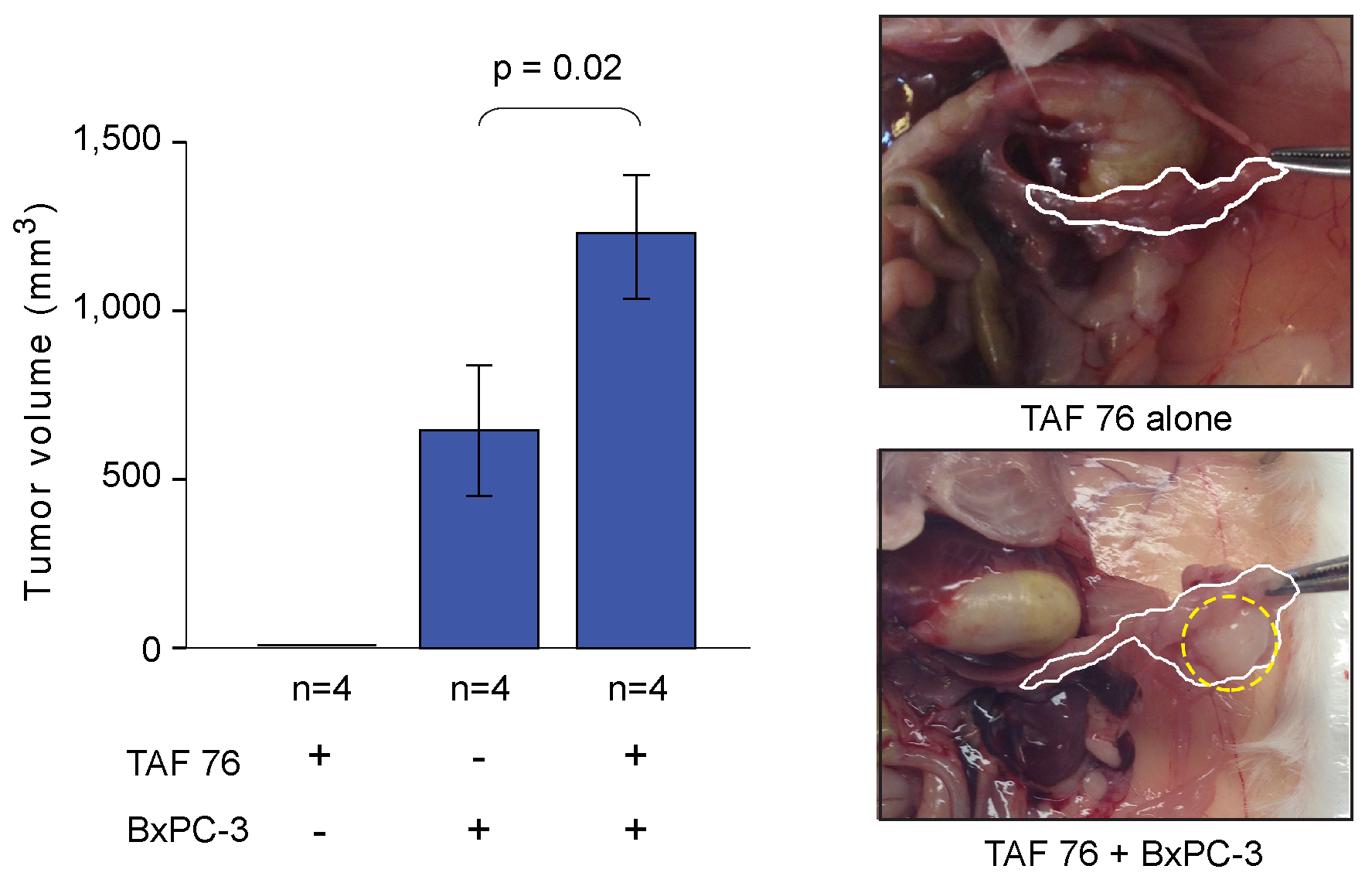

Supplement: Figure S4 — Primary human tumor-associated fibroblasts (TAFs) enhance tumor growth but do not form de novo tumors. Primary pancreatic TAFs were orthotopically injected in NOD/SCID IL2Rγ null mice (3.5×10^5^ cells) with or without BxPC-3 tumor cells (1∶1 ratio). Necropsy at 6 weeks revealed that coinjection of TAFs with tumor cells enhances tumor growth but does not produce a tumor when injected alone. Representative photographs of pancreas (white outline) and tumor (yellow dotted line). (TIF) [file pone.0071978.s004.tif]

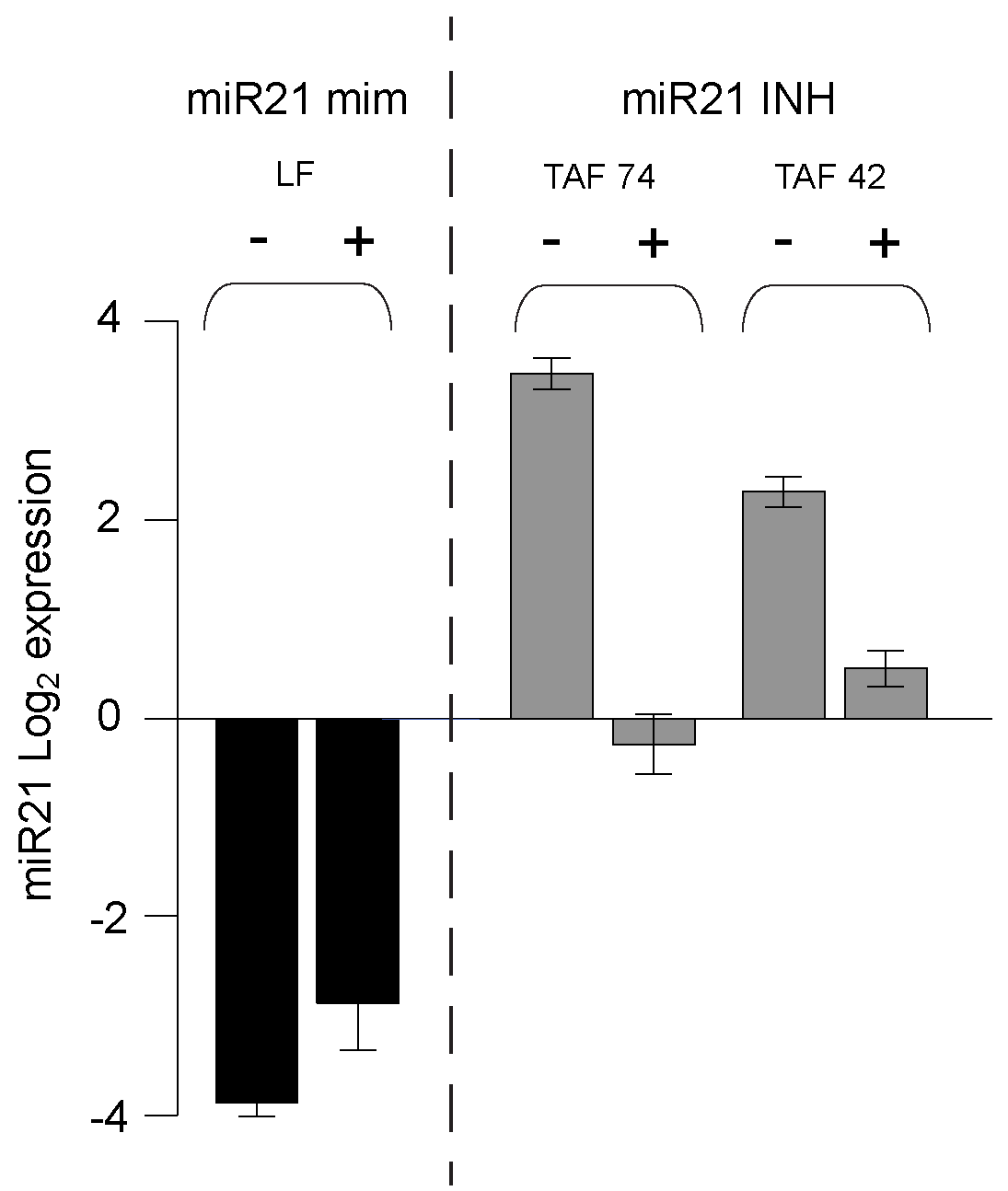

Supplement: Figure S5 — In vitro microRNA-21 overexpression/knockdown. Transfection with miR-21 mimic produces overexpression in normal lung fibroblasts (low baseline miR-21 expression) and anti-sense miR-21 leads to knockdown in primary tumor-associated fibroblast cell lines (TAF) as assessed by qRT-PCR. (TIF) [file pone.0071978.s005.tif]
