# Supplementary material for: MicroRNA-21 in Pancreatic Ductal Adenocarcinoma Tumor-Associated Fibroblasts Promotes Metastasis
Source: PLoS One. 2013 Aug 22;8(8):e71978. doi: 10.1371/journal.pone.0071978 (PMC3750050; doi:10.1371/journal.pone.0071978)
Supplement: Table S1 — TMA baseline patient characteristics. Abbreviations: TMA, tissue microarray, LVI, lymphovascular invasion. (DOCX) [file pone.0071978.s006.docx]

|  |  | **Number (%)** |
| --- | --- | --- |
| **Age (Mean ± SD)** | | 64.2 ± 11.1 |
| **Gender** | Male | 79 (51.6%) |
|  | Female | 74 (48.4%) |
| **AJCC Stage** | IA | 15 (9.8%) |
|  | IB | 26 (17.0%) |
|  | IIA | 29 (19.0%) |
|  | IIB | 78 (51.0%) |
|  | IV | 1 (0.7%) |
| **Lymph Node** | Positive | 69 (45.1%) |
|  | Negative | 79 (51.6%) |
| **LVI** | Positive | 22 (14.4%) |
|  | Negative | 39 (25.5%) |
| **pTumor size** | pT1 | 23 (15.0%) |
|  | pT2 | 63 (41.2%) |
|  | pT3 | 63 (41.2%) |
| **Grade** | Low-mod | 86 (56.2%) |
|  | High | 63 (41.2%) |
| **Margin** | R0 | 128 (83.7%) |
|  | R1 | 19 (12.4%) |
